# Gene expression analysis of embryonic stem cells expressing VE-cadherin (CD144) during endothelial differentiation

**DOI:** 10.1186/1471-2164-9-240

**Published:** 2008-05-22

**Authors:** Vesna Nikolova-Krstevski, Manoj Bhasin, Hasan H Otu, Towia Libermann, Peter Oettgen

**Affiliations:** 1 Division of Cardiology, Department of Medicine, Beth Israel Deaconess Medical Center and Harvard Institutes of Medicine, Boston, USA; 2 Division of Interdisciplinary Medicine and Biotechnology, Department of Medicine, Beth Israel Deaconess Medical Center and Harvard Institutes of Medicine, Boston, USA

## Abstract

**Background:**

Endothelial differentiation occurs during normal vascular development in the developing embryo. This process is recapitulated in the adult when endothelial progenitor cells are generated in the bone marrow and can contribute to vascular repair or angiogenesis at sites of vascular injury or ischemia. The molecular mechanisms of endothelial differentiation remain incompletely understood. Novel approaches are needed to identify the factors that regulate endothelial differentiation.

**Methods:**

Mouse embryonic stem (ES) cells were used to further define the molecular mechanisms of endothelial differentiation. By flow cytometry a population of VEGF-R2 positive cells was identified as early as 2.5 days after differentiation of ES cells, and a subset of VEGF-R2^+ ^cells, that were CD41 positive at 3.5 days. A separate population of VEGF-R2^+ ^stem cells expressing the endothelial-specific marker CD144 (VE-cadherin) was also identified at this same time point. Channels lined by VE-cadherin positive cells developed within the embryoid bodies (EBs) formed by differentiating ES cells. VE-cadherin and CD41 expressing cells differentiate in close proximity to each other within the EBs, supporting the concept of a common origin for cells of hematopoietic and endothelial lineages.

**Results:**

Microarray analysis of >45,000 transcripts was performed on RNA obtained from cells expressing VEGF-R2^+^, CD41^+^, and CD144^+ ^and VEGF-R2^-^, CD41^-^, and CD144^-^. All microarray experiments were performed in duplicate using RNA obtained from independent experiments, for each subset of cells. Expression profiling confirmed the role of several genes involved in hematopoiesis, and identified several putative genes involved in endothelial differentiation.

**Conclusion:**

The isolation of CD144^+ ^cells during ES cell differentiation from embryoid bodies provides an excellent model system and method for identifying genes that are expressed during endothelial differentiation and that are distinct from hematopoiesis.

## Background

A close temporal and spatial association exists between the development of hematopoietic and endothelial stem cells during embryogenesis. This is first observed in extra-embryonic mesodermal tissues such as the yolk sac. Within the developing yolk sac primitive erythrocytes are surrounded by a layer of angioblasts [1,2]. The close temporal and spatial association of these two lineages led to the hypothesis that they arise from a common precursor of mesodermal origin known as the hemangioblast [3]. Further support for the existence of a common precursor comes from gene targeting disruption studies, which demonstrate that some genes are essential for the development of both lineages. For example, the *SCL/tal-1 *gene, previously described for its essential role during hematopoiesis, is also expressed in the developing vasculature and is required for vascular remodeling during embryogenesis [4,5]. One of the earliest cell surface markers that defines this bipotential cell is the VEGF receptor-2 (VEGF-R2). Targeted disruption of VEGF-R2 similarly leads to defects in vasculogenesis and hematopoiesis [6].

One powerful tool to study the earliest steps in the differentiation of hematopoietic and endothelial cells is the use of embryonic stem cells in culture [7]. Most studies suggest that the molecular events leading to the development of the hematopoietic and endothelial lineages in embryoid bodies (EBs) derived from ES cells in culture are very similar to those observed during embryonic development [8,9]. During ES cell differentiation, blast colony-forming cells (BL-CFCs) can be isolated from EBs that exhibit endothelial and hematopoietic potential [8]. BL-CFCs expressing VEGF-R2 can be identified within EBs about 2.5 days.

Several transcription factors that are required for definitive hematopoiesis have been identified (Table 2). The transcription factor *Runx1 *(AML1), a member of the core binding factor (CBF) family of transcription factors is required for the establishment of definitive, but not primitive hematopoiesis [10]. The transcription factor GATA-1 is required for differentiation of the erythroid lineage [11]. Finally, the homeobox transcription factor HoxB4 was shown to promote the potential of hematopoietic stem cells derived from ES cells that contribute to definitive hematopoiesis, promoting the development of self-renewing, long term, hematopoietic progenitors [12].

Considerably less is known about the transcription factors and the molecular mechanisms that regulate endothelial differentiation. The purpose of this study was to use an ES cell differentiation system to identify genes that are preferentially expressed at early and later stages of endothelial differentiation. We identified four populations of cells; cells expressing VEGF-R2 (day 2.5), CD41 expressing cells (day 3.5), cells expressing CD144 (VE-cadherin, day 3.5), and cells expressing CD144 (day 6.5). As negative control we have also isolated VEGF-R2 negative (day 2.5), CD41 negative (day 3.5), negative CD144 (VE-Cadherin, day 3.5), and negative CD144 (day 6.5). In addition, we isolated CD144 positive and CD144 negative cells from mouse embryos at embryonic day 9.5. Microarray analysis of the RNA isolated from each of these populations of cells allowed for the identification of genes expressed in these subsets of cells and suggest their potential role during the differentiation of embryonic stem cells along the endothelial lineage.

## Methods

### ES cell culture

CCE Embryonic stem cells (ATCC) were maintained on irradiated primary embryonic fibroblasts (Chemicon) in knockout DMEM (Invitrogen/Gibco-BRL) supplemented with 15% fetal bovine serum (Hyclone, Logan, UT), penicillin/streptomycin 1% (Invitrogen/Gibco-BRL), l-glutamine 2 mM (Invitrogen/Gibco-BRL), non-essential amino acids 0.1 mM, nucleosides 0.1 mM, 2-mercaptoethanol 0.1 mM (Sigma, St. Louis, MO), monothioglycerol (MTG) 2 mM (Sigma), and ESGRO leukemia inhibitory factor (LIF) 1000 units/ml (Sigma). ES cells were grown in LIF-containing media for 48 hours. To generate EBs, ES cells were separated from the feeder cells, re-plated at a density of 2 × 10^6 ^cells per 10 cm dish (Fisher Scientific), and grown in media without LIF to promote differentiation.

### Flow Cytometry and Cell Sorting

To prepare single-cell suspensions EBs were washed with PBS and disrupted by pipetting after 20 min treatment with cell dissociation solution at 37°C (Sigma). Whole embryo cells were dissected from the decidual tissue of E9.5 pregnant 129/Sv mice and washed in PBS (Invitrogen). Dissected embryos were incubated for 60–90 minutes at 37°C in 0.1% collagenase/dispase (Sigma) and 20% fetal bovine serum (FBS, Hyclone) and subsequently dissociated into a single-cell suspension. All cells were fixed in 3:1 ethanol:glacial acetic acid and washed extensively in PBS prior to immunofluorescent staining. Cells were initially incubated with rat-monoclonal anti-VE-cadherin antibody (1:100, Pharmingen, San Diego, CA) at room temperature for 30 min, followed by labeling with anti-rat fluorescein-isothiocynate (FITC)-conjugated antibody (1:100, Jackson Laboratories). Subsequent double labeling with phycoerythrin (PE)-conjugated anti-VEGF-R2 and -CD41 antibodies (1:100, Pharmingen) was also performed at room temperature for 30 min. Flow cytometry and cell sorting was completed on the FC5000 Flow Cytometer at the Beth Israel Deaconess Medical Center Flow Cytometry Core Facility using CXP Analysis Program.

### RNA Extraction and Quantitative RT-PCR

Total RNA from FACS-sorted cells was isolated using an RNeasy kit (Qiagen). Real-time PCR was performed containing SYBR green I (1:1000 dilution, Molecular Probes), forward and reverse primers (0.8 μM each), 0.8 mM dNTPs, 2 mM MgCl_2_, 1U Taq Polymerase (Promega), and 1U Platinum Taq Antibody (Invitrogen) in 1xPCR Buffer under the following conditions: denaturation at 95°C (3 min); 35 cycles at 94°C (1 min), at 60°C (1 min), at 72°C (1 min). GAPDH was used as an internal reference in each reaction. Amplification was followed by melting curve analysis using the program run at the step acquisition mode to verify the presence of a single amplification product in DNA. Accumulation of PCR products was monitored and determined using the Opticon Monitor (MJ Research). The threshold cycle (C_T_) was determined using the Opticon analysis software. Oligonucleotide Primers for Quantitative RT-PCR for each gene were:

Ikaros: Forward GCCCTATGACAGTGCCAACT, Reverse CAGCTGGTACATGGAGCTGA

AML1: Forward TGTTGGGCATTTGACTTTGA, Reverse TTACTACCGGAGGGTTGTGG

Hemoglobin Y: Forward AAGCTCCGAGCACACCCACT, Reverse AAGCTCTGAGCACACCCACT

CD41: Forward AAGCTCTGAGCACACCCACT, Reverse CTCAGCCCTTCACTCTGACC

Endothelin Receptor B: Forward CAGGAAGAAGAGCGGTATGC, Reverse CACACCTGTGTGGATTGCTC

Thrombospondin: Forward CCAAAGCCTGCAAGAAAGAC, Reverse CCTGCTTGTTGCAAACTTGA

Tie1: Forward CAGGCACAGCAGGTTGTAGA, Reverse GTGCCACCATTTTGACACTG

Tie2: Forward AAGCATGCCCATCTGGTTAC, Reverse GTAGGTAGTGGCCACCCAGA

EphrinA1: Forward CCCACATTACGAGGACGACT, Reverse CCCAAGCTAAAAGGCCTCAA

Flt-1: Forward CCAAGGCCTCCATGAAGATA, Reverse ATACTGTCAGGGGCTGGTTG

MEF2C: Forward ACGCCTGTCACCTAACATCC, Reverse AGCTCTCAAACGCCACACTT

KLF2: Forward CCAAGAGCTCGCACCTAAAG, Reverse GTGGCACTGAAAGGGTCTGT

### Microarray analysis

For transcriptional profiling, the mouse genome 430 2.0 Affymetrix GeneChip, containing more than 45,000 transcripts was used. RNA for the microarray experiments was obtained in duplicates from two separately conducted experiments using the murine ES cells. Microarray analysis was conducted via the Genomics Center at the Beth Israel Deaconess Medical Center, according to previously described protocols for total RNA extraction and purification, cDNA synthesis, *in vitro *transcription reaction for production of biotin-labeled cRNA, hybridization of cRNA with mouse genome 430 2.0 Affymetrix gene chips, and scanning of image output files [13]. The quality of the scanned array images was determined on the basis of background values, percent present calls, scaling factors, and 3'-5' ratio of β-actin and GAPDH using the BioConductor R packages [14].

Scanned array images were analyzed by dChip, as it is more robust than MAS5.0 and RMA in signal calculation. The raw probe level data was normalized using smoothing-spline invariant set method. The signal value for each transcript was summarized using perfect-match-only (PMO) based signal modeling algorithm described in dChip. The PMO based modeling algorithm yields less number of false positives as compared to the PMO-MM model (MM meaning "mismatch-match"). In this way, the signal value corresponds to the absolute level of expression of a transcript [15]. These normalized and modeled signal values for each transcript were used for further high-level bioinformatics analysis. During the calculation of model based expression signal values, array and probe outliers are interrogated and images spike are treated as signal outliers. The outlier detection was carried out using dChip outlier detection algorithm. A chip is considered as an outlier if the probe, single or array outlier percentage exceeds a default threshold of 5%.

When comparing two groups of samples to identify genes enriched in a given phenotype, if 90% lower confidence bound (LCB) of the fold change (FC) between the two groups was above 2.0, the corresponding gene was considered to be differentially expressed [16]. LCB is a stringent estimate of FC and has been shown to be the better ranking statistic [15]. It has been suggested that a criterion of selecting genes that have a LCB above 2.0 most likely corresponds to genes with an "actual" fold change of at least 3 in gene expression [16,17].

The final list of positive, phenotype-specific, differentially expressed genes was generated by removing all the genes that were also differentially expressed in the negative control population during the same transition. For example, the initial list of differentially expressed genes for VEGF-R2^+^/CD41^+ ^at day 3.5 as compared to VEGF-R2^+ ^at day 2.5 was corrected by removing the genes that are also differentially expressed in the negative control cells (VEGF-R2^-^/CD41^- ^at day 3.5 compared to VEGF-R2^- ^at day 2.5). Further corrections in the identified differentially expressed genes were made by comparing changes in the expression in negative populations (e.g. CD144-) versus positive populations (e.g. VEGF-R2^+ ^at day 2.5) over time.

The raw MIAME compliant microarray data is available online in the GEO repository at NCBI [18].

### Western Blot Analysis

Whole cell lysates (50 μg) of cells were boiled in Laemmli buffer and resolved on 10% SDS-PAGE acrylamide gel. Proteins were transferred on Hybond-PVDF membranes and immunoblotted using the ECL Western Blotting Detection reagents (Amersham Biosciences, UK). Polyclonal antibodies against VE-cadherin, CD41, and alpha actin were obtained from Pharmingen.

### Immunohistochemistry and microscopy of embryoid bodies and the yolk sac

EBs from different time points (day 6.5 to 10.5) were washed with PBS and embedded in OCT freezing compound (Miles Scientific, Cambridge, ML) after fixation in 4% paraformaldehyde for 1 hour at 4°C and cryoprotection in 20% (wt/vol) sucrose overnight. Frozen sections (8–10 μm thick) were mounted onto gelatin-coated glass slides, air-dried overnight, permeabilized for 3 minutes with 0.5% Triton X-100, and washed two times with PBS. Slides were then processed for immunofluorescence microscopy using FITC-conjugated CD41, PE-conjugated VE-cadherin, and FITC-vWF rat antibodies (1:100, Pharmingen). 129/sv mice were bred to generate day E8.5 embryos. The yolk sac was dissected from the embryo proper and fixed in 4% paraformaldehyde, and frozen in OCT. Immunohistochemistry was performed using 10 μm sections. Fluorescent images were obtained using a Nikon Eclispse E800 microscope (Nikon, Tokyo, Japan) equipped with a 40 X/0.75 numeric aperture objective lens as well as a Zeiss AxioCam digital camera (Carl Zeiss International, Heidelberg, Germany). Images were analyzed using Openlab imaging software version 3.0.4 (Improvision, Lexington, MA).

## Results

**Figure 1:**
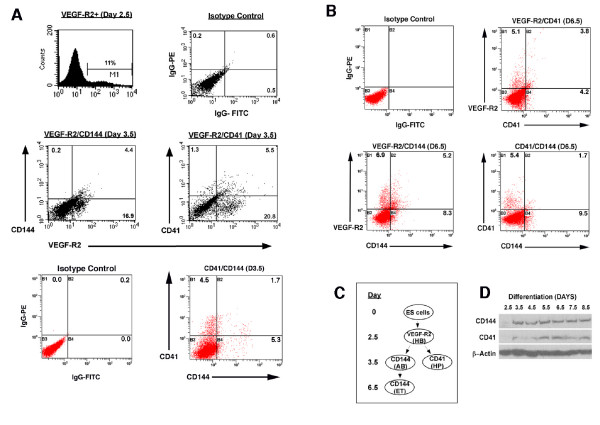
**CD41 and CD144 during ES cell differentiation**. (**A**) Flow cytometric analysis of stem cell differentiation. CCE murine ES cells differentiated into cystic embryoid bodies after removal of LIF (see methods). Flow cytometry was used to determine the expression of VEGF-R2, CD144, and CD41. VEGF-R2 positve cells were first detected at 2.5 days after the removal of LIF and represent approximately 11% of the cells. CD144 and CD41 are first detected at day 3.5. Both markers, CD144 and CD41, represent a subset of VEGF-R2 positive cells (4.4 and 5.5%, respectively). Only a small percentage of CD144 positive cells (1.7%) co-express CD41 at day 3.5 (bottom right panel). Isotype controls are shown in the upper right panel. (**B**) CD144 and CD41-expressing cells remain a subset of VEGF-R2 positive cells (5.2 and 3.8%, respectively) at day 6.5, however, at this time point, the two cell markers are expressed on different cell populations (lower right panel). (**C**) A schematic of ES cell differentiation demonstrating the populations of cells isolated for microarray analysis. VEGF-R2 is a marker of the hemangioblast (HB). CD41 is one of the earliest markers for the hematopoietic progenitor (HP) cells and can be detected as early as day 3.5, concurrently with the endothelial-specific marker CD144 (VE-cadherin) expressed on the angioblast (AB) at day 3.5, and also on differentiated endothelial cells (ET) at day 6.5. (**D**) Protein expression of CD144 (VE-cadherin) and CD41 in embryonic stem cell differentiation. Isolated proteins from developing stem cells on days 2.5 to 8.5 were separated on a 10% SDS-PAGE. Western blot analysis was performed with antibodies directed against CD144, CD41, and β-actin.

The molecular mechanisms underlying endothelial differentiation from pluripotent stem cells remain incompletely understood. We have used a murine ES cell differentiation system to identify the genes involved in the process of endothelial differentiation. Cells were grown in the presence of LIF, and allowed to differentiate into cystic EBs in the absence of LIF. VEGF-R2 positive cells were first detected in ES cells by flow cytometry 2.5 days after the initiation of differentiation (Figure 1A). Approximately 11% of the ES cells expressed VEGF-R2 at this time point. One day later (day 3.5) 20–25% of the ES cells express VEGF-R2. We identified a small subset (4.4%) of VEGF-R2 expressing cells (Figure 1A, middle left) that express the endothelial-cell specific cell surface antigen CD144 (VE-cadherin) at 3.5 days. One of the earliest cell surface markers of the hematopoietic lineage has recently been identified as CD41 (glycoprotein IIb), which is first detected in differentiating ES cells at 3.5 days [19]. Approximately 5% of cells express CD41 and VEGF-R2 at this time point (Figure 1A, bottom right). At day 6.5, CD41 and CD144 are still expressed on a subset of VEGF-R2 positive cells (3.8 and 5.2%, respectively). However, the individual expression of CD41 and CD144 seems to diverge onto separate cell populations, with only a small percentage (1.7%) of CD144 positive cells showing CD41 co-expression (Figure 1B, lower right panel). Also, by day 6.5, the number of VEGF-R2 expressing-cells is significantly decreased (approximately 11% at day 6.5, compared to 20% at day 3.5) (Figure 1B).

VE-cadherin is expressed on the surface of endothelial cells, during vascular development and in the adult cells [20]. To verify the expression of these cell surface markers at the protein level, western blot analysis was performed at different time points during ES cell differentiation. No expression of CD41 or CD144 was observed at 2.5 days (Figure 1D). However, at day 3.5 there was a significant increase in the expression of both CD144 and CD41.

**Figure 3:**
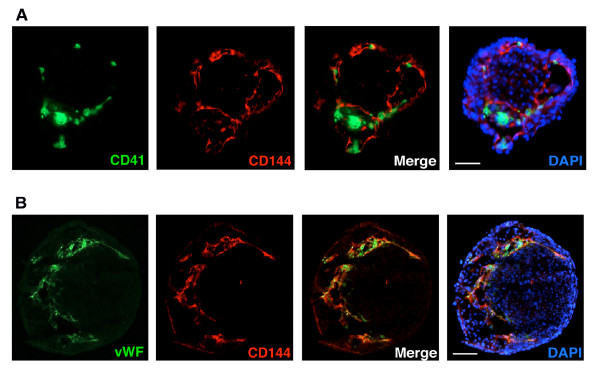
**Expression of CD41 and CD144 in embryoid bodies**. Immunohistochemical analysis of CD41 and CD144 staining of frozen embryoid body sections (see methods for details). (**A**) Evaluation of CD41 (green) and CD144 (red) staining by immunofluorescence in embryoid body at day 6.5. Overlapping images of CD41 and CD144 demonstrate spatial proximity of CD41/CD144 expression. DAPI nuclear staining (blue) outlines the general embryoid body morphology and organization. Bar represents 300 μm. (**B**) Immunofluorescence staining for vWF (green), VE-Cadherin (red), and DAPI (blue) in embryoid bodies at day 10.5. Colocalization of the two endothelial-specific markers, vWF and CD144, is evident in the merged images. Bar represents 200 μm.

**Figure 4:**
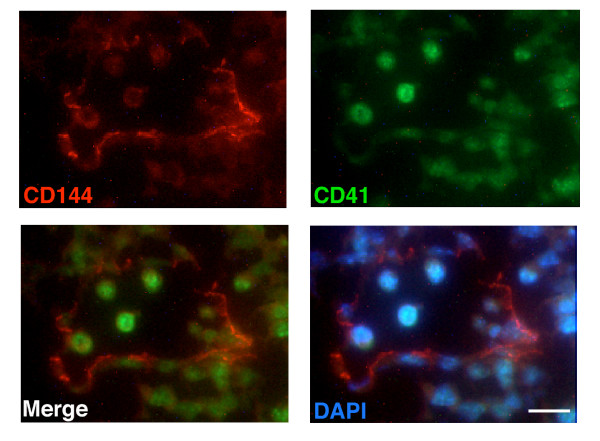
**Expression of CD41 and CD144 in the yolk sac**. Immunostaining of CD144 and CD41 within the yolk sac of E8.5 embryos (see methods for details). Similar to the staining pattern observed in the embryoid body, a close proximity of CD144 (red) and CD41-positive cells (green) is also observed in the yolk sac. Bar represents 100 μm; see methods for details of microscopy.

Because of the close temporal and spatial relationship between hematopoiesis and endothelial differentiation in the developing embryo, we were also interested in evaluating the spatial relationship between the expression of CD41 and CD144 in the EBs. We observed the formation of small channels within the EBs lined by CD144 positive cells in 6.5 day EBs (Figure 3A). CD41 expressing cells (green) were observed in discrete sections of the EBs, and appeared to be located within the lumen of the channels formed by the CD144 positive cells (red). Nuclear staining with DAPI demonstrated that the areas where CD41 and CD144 are expressed represent distinct regions within the EBs (Figure 3A). Co-localization of CD41 and CD144 positive cells further validated the close proximity of cells expressing these two markers. To further validate the endothelial cell specific nature of the CD144 positive cells, the expression of the endothelial cell specific marker von Willebrand's factor (vWF) was also evaluated. The expression pattern of CD144 and vWF observed in the EBs is nearly identical (Figure 3B). To extend these studies we also evaluated the expression of CD144 and CD41 in the developing yolk sac. We observed a close association between the expression of CD144 and CD41 in the yolk sac, with the lumen of blood vessels in the yolk sac lined by CD144 positive cells, and CD41 positive cells within the lumen (Figure 4). These studies confirm the close temporal and spatial association between the differentiation of endothelial cells and hematopoietic cells from pluripotent stem cells in the developing embryo, and support the ability of murine ES cells to recapitulate the steps associated with hematopoietic and endothelial differentiation, using an *in vitro *differentiation model.

**Figure 2:**
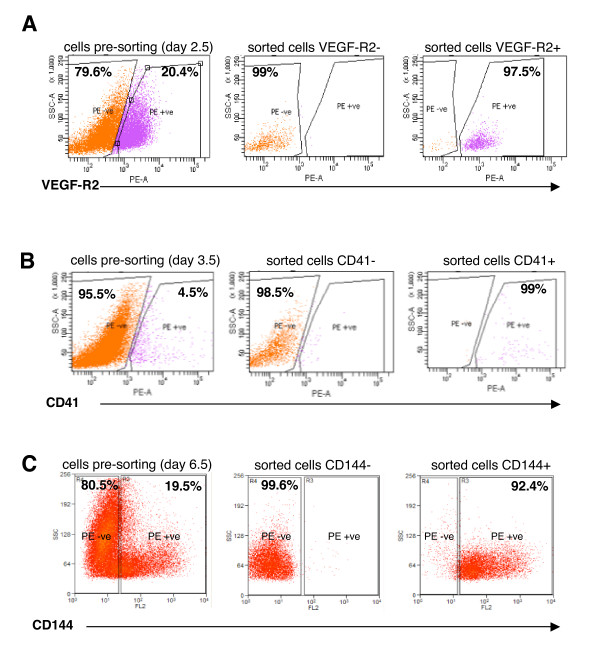
**Purity controls for FACS sorting**. Dot-plots in this figure show EB cells labeled with (**A**) VEGF-R2 antibody at day 2.5, (**B**) CD41 at day 3.5, and (**C**) CD144 marker at day 6.5. The plots on the left show total cell populations labeled with the specific cell marker before FACS sorting, while the plots in the middle and right are the purity controls demonstrating the negative and positive cell populations, respectively, after FACS sorting.

To begin to define the molecular mechanisms underlying the differentiation of hematopoietic and endothelial cells from a bipotent hemangioblast, we performed microarray experiments using RNA derived from different subsets of ES cells at different time points during the process of differentiation. The four positive populations isolated (Figure 1C), included VEGF-R2 positive population at day 2.5 (also referred to as hemangioblast (HB)), CD41 positive population at day 3.5 (also referred to as hematopoietic precursor (HP)), CD144 positive population at day 3.5 (also referred to as the angioblast (AB)), and CD144 positive population at day 6.5 (also referred to as endothelial cell (ET)). Additionally, as negative control cells, we have also isolated four negative populations, VEGF-R2 negative population at day 2.5 (NHB), CD41 negative population at day 3.5 (NHP), CD144 negative population at day 3.5 (NAB), and CD144 negative population at day 6.5 (NET). Microarray analysis was performed using Affymetrix Mouse Genome 430 2.0 chips at the Beth Israel Deaconess Medical Center Microarray facility. Dot plots in Figure 2 demonstrate the efficiency of the FACS sorting and purity of each of the isolated cell populations.

### Early VEGF-R2 positive ES cells express several mesodermal markers

One of the earliest markers of the bipotent hemangioblast is VEGF-R2. These cells are of mesodermal origin, and have previously been shown to express mesodermal markers such as *brachyury *[3]. We examined the expression of genes in the VEGF-R2 positive population of cells at day 2.5, compared to those at later stages of ES cell differentiation such as VEGFR2^+^/CD41^+ ^and VEGFR2^+^/CD144^+ ^(Table 1,2,3,4,5,6,7). In addition to *brachyury*, we identified several other genes, including the homeobox genes *lim1*, *Mixl1*, *even skipped*, and *goosecoid*, all of which are highly expressed in the embryonic mesoderm (Table 1). These results support the idea that the VEGF-R2 positive cells, isolated at day 2.5 are of mesodermal origin and undergo differentiation in this model system.

**Table 1:** Mesodermal Markers Up-regulated in VEGF-R2 positive cells at day 2.5 of ES Cell Differentiation*

**Gene Bank accession**	**Gene**	**HBvsHP**	**HBvsAB**
AF154573	Mix1 homeobox	22.43	21.00
AV335209	Lim homeobox protein 1	10.11	13.33
NM_009309	Brachyury	9.90	12.01
NM_007966	Even skipped homeotic gene	13.82	14.28
NM_010351	Goosceoid	9.44	10.46

**Table 2:** Comparison of CD41-postive (day 3.5) to VEGF-R2 positive cells (day 2.5)*

**Gene Bank Accession number**	**Gene**	**Fold Change**
M26898	Hemoglobin X	266.22
AV311770	Hemoglobin Z	119.48
AV156860	Hemoglobin Y	39.49
NM_010369	Glycophorin A	18.76
AK010968	Erythropoietin receptor	5.99
NM_013706	CD52	5.98
NM_010824	Myeloperoxidase	5.06
NM_053149	Hemogen	7.72
NM_010635	Krueppel like factor-1	24.81
NM_008089	GATA 1	22.52
AV317621	Ikaros transcription factor	7.51
BB319935	Helios transcription factor	6.26
BB795285	AML-1	5.18

**Table 3:** Comparison of CD144-postive (day 3.5) to VEGF-R2 positive cells (day 2.5)*

**Gene Bank Accession number**	**Gene**	**Fold Change**
BF100813	Endothelin receptor type B	4.12
NM_011587	Tie1	5.49
NM_031198	Transcription factor EC	4.02
NM_008452	Krueppel like factor 2	6.12
BB331017	Sox11	5.01
BM220880	Forkhead box P1	4.87

**Table 4:** Comparison of CD144-postive (day 6.5) to VEGF-R2 positive cells (day 2.5)*

**Accession Number**	**Gene**	**Fold Change**
BF100813	Endothelin receptor type B	19.07
D38146	Ephrin A1	5.53
NM_011587	Tie1	8.82
NM_008816	PECAM-1	8.87
NM_013805	Claudin 5	10.50
BC020129	Robo4	3.55
AY083458	CD 109 antigen	3.36
AI595932	Mef2C	5.24
BB331017	Sox11	5.36
NM_008452	KLF2	4.78

**Table 5:** Comparison of embryonic CD144-postive to CD144-negative cells (day 9.5)*

**Accession number**	**Gene**	**Fold Change**
NM_010494	ICAM-2	1614.76
NM_011587	Tie1	239.47
AW543698	CD144	84.44
AV319507	Multimerin 2	67.79
NM_007932	Endoglin	106.71
NM_010228	Flt-1	50.20
BB667216	Von Willebrands Factor	56.07
NM_008816	PECAM-1	31.40
NM_008713	Endothelial nitric oxide synthase	20.83
D43775	Endothelin-1	22.59
D38146	Ephrin A1	13.80
NM_009378	Thrombomodulin 1	8.77
BC020129	Robo 4	7.25
AK004781	Sox 17	34.96
AV329219	ERG	228.66
NM_009236	Sox 18	20.64
BB831146	C/EBP delta	8.96
AI595932	MEF2C	5.17
AK004675	GATA2	4.26
NM_008452	KLF2	3.24
NM_016791	NFATc1	3.27
BC005686	ELK3	5.15
AI462296	Forkhead box 01	2.67

**Table 6:** Genes Enriched in all CD144-positive cells

**Gene**	**Fold Change (vs. CD144-E9.5)**	**(d 6.5 vs HB)**	**(d 3.5 vs HB)**
CD34	304.4	8.78	3.0
Tie1	239.5	6.82	2.98
Endomucin	21.72	11.35	4.07
Ephrin A1	13.8	7.87	2.99
TFEC	8.7	4.56	4.02
KLF2	3.24	3.66	3.69

**Table 7:** Genes that showed increased expression in CD144-positive cells from EBs (day 6.5) and in the embryo (day 9.5) compared to CD41-positive (day 3.5) and CD144-negative cells (day 6.5) from Ebs

**Accession number**	**Gene**	**(EC vs HB)**	**(EC vs CD144-cells)**
NM_010494	ICAM-2	3.37	1614.76
AW543698	CD144	3.65	84.44
AV319507	Multmerin 2	3.83	67.79
NM_016900	Caveolin2	5.49	114.87
NM_013805	Claudin 5	3.76	36.04
D38146	Ephrin A1	7.87	13.80
AY083458	CD109 antigen	3.36	9.83
BC020129	Robo 4	3.55	7.25
NM_007585	Annexin A2	6.0	7.25
NM_008816	PECAM-1	6.5	31.40
AI595932	MEF2C	6.86	5.17
NM_011441	Sox 17	4.14	34.96

* The values shown in the columns are corrected fold changes (FC). The corrected FC for a transcript is obtained by subtracting the FC for the particular transcript in the negative control populations from FC for the positive populations (see methods for details).

### Markers of Hematopoiesis in the CD41 expressing ES cells at day 3.5

We identified a small population of VEGF-R2 positive cells expressing the early hematopoietic marker CD41 and cells not expressing CD41 (negative control) at day 3.5. Microarray analysis was performed on RNA derived from these cells. VEGFR2^+^/CD41^+ ^compared to the VEGF-R2 positive cells at day 2.5 have 565 differentially expressed transcripts. Out of these 565 transcripts, all the transcripts that were differentially expressed in the negative control cells in the same transition (VEGF-R2^-^/CD41^- ^cells at day 3.5 as compared to VEGF-R2^- ^cells at day 2.5 or VEGF-R2^-^/CD41^- ^cells at day 3.5 as compared to VEGF-R2^+ ^cells at day 2.5) were filtered out. The final list of VEGFR2^+^/CD41^+ ^specific differentially expressed transcripts consists of genes associated with hematopoiesis (Table 2). It has also been suggested that *in vitro *ES cell differentiation systems closely recapitulates the events associated with hematopoietic development in the yolk sac, including primitive erythropoiesis, megakaryocyte, and mast cell differentiation [21]. The first of these to occur is primitive erythropoiesis. We identified transcription factors upstream of erythropoiesis, including AML-1, GATA-1, and erythroid Krueppel-like factor (KLF1), and genes predominantly associated with the erythroid lineage (Table 2). However, Ikaros and Helios, two zinc finger transcription factors known to regulate lymphopoiesis, were also enriched in this CD41-positive population [22,23]. Helios expression is restricted to cells of T cell origin. These results suggest that either these transcription factors exhibit additional functions at earlier stages of hematopoiesis or that initiation of other hematopoietic lineages, in addition to the erythroid lineage, occurs during ES cell differentiation.

### Markers of endothelial differentiation expressed in CD144 positive cells

To evaluate whether genes of the endothelial lineage are expressed in early CD144 (VE-cadherin) positive cells during ES cell differentiation, we compared microarray data of RNA isolated from CD144 positive cells at day 3.5 to VEGF-R2 positive cells at day 2.5. To identify genes that are uniformly upregulated in the CD144 positive cells, we also performed microarray analysis of RNA samples from the negative controls (i.e CD144 negative cells at day 3.5 to VEGF-R2 negative cells at day 2.5). The CD144 positive cells at day 3.5 have 483 differentially expressed transcripts that are further corrected by removing the transcripts differentially expressed in the negative control cells in the same transition (CD144^- ^day 3.5 vs. VEGF-R2^- ^day 2.5) or (CD144^- ^day 3.5 vs. VEGF-R2^+ ^day 2.5). This final list has enrichment of selected endothelial-specific genes, including Tie1 and the endothelin receptor B (Table 3). In addition, several transcription factors were identified as being enriched at this stage, including Krueppel-like factor 2 (KLF2), the SRY-box (Sox) 11, and the Forkhead box P1 transcription factor.

To examine the specific gene expression profile at later stages of endothelial differentiation, we performed microarray analysis of CD144 positive and CD144 negative cells isolated from ES cells 6.5 days after differentiation. The CD144 positive cells at day 6.5 compared to VEGF-R2 positive cells obtained at day 2.5 showed differential expression of 1068 transcripts. Transcripts that were differentially expressed in the negative control cells (CD144^- ^day 6.5 vs VEGF-R2^- ^day 2.5 and CD144^- ^day 6.5 vs VEGF-R2^+ ^day 2.5) were removed from the list. Additional analysis was performed to eliminate remaining false positive genes (see methods). The final list has several endothelial-specific genes including PECAM-1, claudin 5, and roundabout homolog 4 (Robo 4) (Table 4).

To identify genes that are selectively enriched in endothelial cells during embryonic vascular development, we also isolated CD144 positive cells from murine embryos at day 9.5. CD144 positive cells were sorted and separated from CD144 negative cells, and microarray analysis was performed. Because the number of CD144 positive cells isolated from the embryos was much smaller, an amplification step was performed prior to the microarray analysis. The results of the microarray analysis also demonstrate an enrichment of several endothelial-specific genes including ICAM-2, VE-cadherin, PECAM-1, and endothelial nitric oxide synthase (eNOS) (Table 5). The expression of several transcription factors was also determined to be enriched in this population of cells, including SOX 17, the Ets factor Erg and Elk3, Vezf1, Foxo1, and C/EBP delta. Table 6 shows a condensed list of genes whose expression is increased in all CD144 positive cells, including the ones isolated at different stages of EB differentiation (day 3.5 and 6.5), and day 9.5 embryos. The genes upregulated in the CD144 positive cells were not detected in hematopoietic cells (CD41 positive cells at day 3.5) (Table 7).

**Figure 5:**
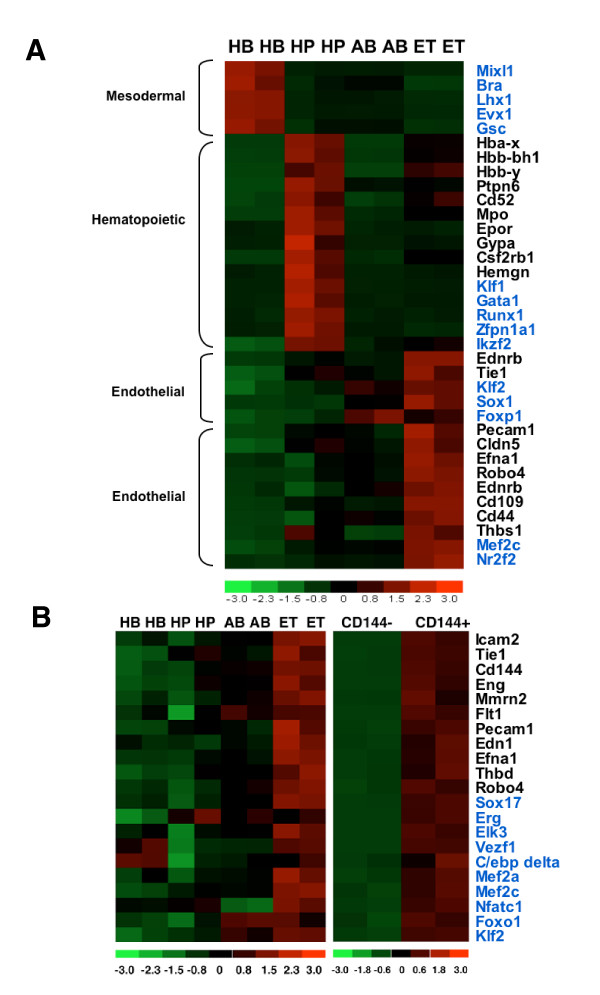
**Heat map of selected genes at different stages of ES cell differentiation and embryogenesis**. (**A**) Expression of selected mesodermal, hematopoietic, and endothelial genes expressed in VEGF-R2 positive (HB), CD41-positive (HP), and CD144-positive (AB and ET) murine ES cells. (**B**) Expression of selected genes identified as being enriched in CD144^+ ^cells in the developing mouse embryo at day E9.5 compared to CD144-cells. Transcription factors are denoted with a blue color. Signal values are normalized within the [-3,3] interval as shown by the color scale at the bottom of the image where red indicates high expression and green indicates low expression.

A color map of the relative expression levels obtained via microarray analysis, including the mesodermal, hematopoietic, early and late endothelial markers was also generated (Figure 5). Signal values were normalized within the [-3,3] interval as shown by the color scale at the bottom of the image where red indicates high expression and green indicates low expression.

**Figure 6:**
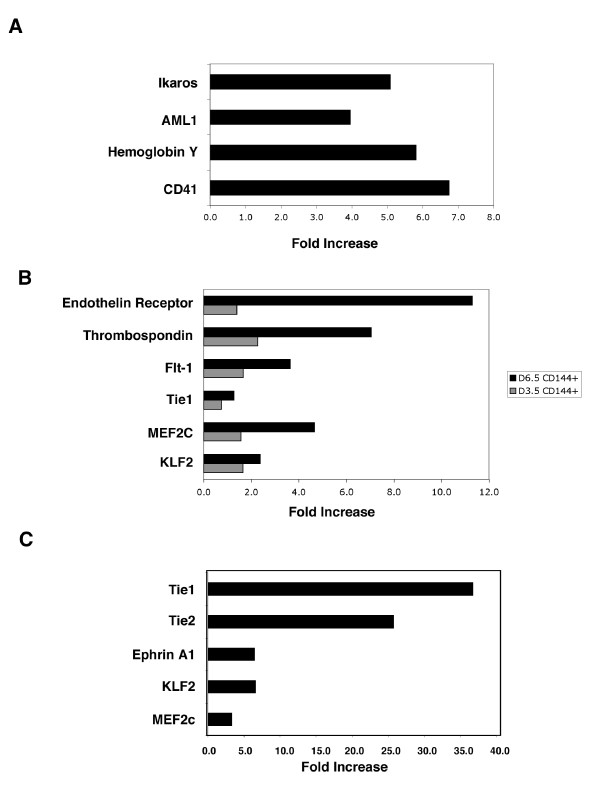
**Quantitative RT-PCR of selected genes identified by microarray analysis**. (**A**) Fold induction, as measured by quantitative RT-PCR, of the expression of representative genes identified as being enriched in CD41 positive cells (day 3.5) compared to VEGF-R2 positive, CD41 negative cells at day 2.5 (Table 1B). (**B**) Fold induction of representative genes in CD144 positive cells at day 3.5 (gray bars) and day 6.5 (black bars) compared to VEGF-R2 positive, CD144 negative cells at day 2.5 (Table 1C and D). (**C**) Fold induction of a subset of genes identified in CD144 positive versus CD144 negative cells in the developing mouse embryo at day E9.5, as measured by quantitative RT-PCR (Table 1E).

The expression of a representative number of the genes identified to be enriched in the CD41 and CD144 expressing stem cells (Table 1,2,3,4,5,6,7) were also evaluated by quantitative RT-PCR. The results of these studies confirm the expression patterns observed in each of the populations observed by microarray analysis (Figure 6). For example, a subset of the genes identified to be enriched in the CD41 positive cells (day 3.5) compared to VEGF-R2 positive cells (negative for CD41) at day 2.5 (Table 2) were evaluated by quantitative RT-PCR. These genes, including Ikaros, AML-1, hemoglobin Y, and CD41, and are expressed 4–7 fold higher in the CD41 positive cells (Figure 6A). Similarly a number of genes found to be upregulated in CD144 positive cells at day 3.5 and 6.5 during ES cell differentiation compared to VEGF-R2 positive cells (negative for CD144) (Table 3 and 4), were similarly validated by quantitative RT-PCR (Figure 6B). Many of these genes, including the endothelin receptor, Flt-1, and Tie1, are known to be endothelial cell specific, or involved in regulating endothelial function. The expression of a similar set of genes was also enriched in CD144 positive versus CD144 negative cells in the mouse embryo at day E9.5 (Table 5), a subset of which, including Tie1, Tie2, Ephrin A1, KLF2, and MEF2C, were validated by quantitative RT-PCR (Figure 6C).

To further define the genes that are upregulated at the angioblast stage (day 3.5 CD144 positive), and later stages of endothelial differentiation in the EBs (day 6.5 CD144 positive), and in CD144 positive cells of the embryo (day 9.5), we performed a comparative analysis of these genes (on-line Additional file 1). We identified 41 genes (section VI) that are enriched in all three populations, 139 genes that are enriched in CD144 positive cells of the EB at early and later stages (Section IV), and 119 genes (Section V) that are enriched in CD144 positive cells in later stages of the EB (day 6.5) and in CD144 positive cells of the embryo at day E9.5. Some of the genes identified in the overlapping sections of the Venn diagram are listed in Table 6 and 7. A complete list of the up and down-regulated genes are provided in an on-line supplement [24].

In addition to identifying genes that are enriched at various stages of differentiation, we were also interested in determining whether the genes identified have previously been shown to be linked to each other, or to other genes with regard to specific pathways. In the past few years a number of bioinformatic programs that are designed to evaluate potential links between the genes identified and potential pathways have been developed. We used the program PathwayAssist (Ariadne Genomics Inc.) to evaluate the potential of underlying existing pathways at various stages of endothelial and hematopoietic development. In this approach a natural language processing module, MedScan, is used to describe events of regulation, interaction and modification between proteins, cell processes and small molecules and the results are generated in the forms of biological pathways, gene regulation networks and protein interaction maps [25]. Because considerably more is known regarding the genes involved in hematopoiesis, we first performed analysis of the genes enriched in the CD41 positive cells. The initial analysis was performed using transcription factors identified in the CD41 positive cells at day 3.5 in the differentiating EBs compared to the VEGF-R2 positive cells at day 2.5. When the transcription factors alone are entered into this pathway analysis, the specific factors are linked in multiple ways. For example, KLF1 and GATA1, which are known to be involved in erythropoiesis cluster together (Additional file 2) with links to references for pathways). Similarly, Ikaros, Aiolos, and Helios, zinc finger transcription factors, are involved in lymphopoiesis are linked together. AML1 (Runx1) is known to be upstream of all of the hematopoietic lineages. The pathway analysis was further expanded to include other proteins that are enriched in the CD41 positive population, including the hemoglobin genes, myeloperoxidase, c-kit, glycophorin, and other genes, in addition to the transcription factors identified. Although, significantly more complex, the pathway analysis similarly supports the linkage of these transcription factors and pathways with specific target genes along various hematopoietic lineages (on-line Additional file 3). In addition to demonstrating the utility of this kind of pathway analysis for linking transcription factors with known gene targets and pathways that have previously been defined for hematopoiesis, these results also support the potential of this approach for identifying similar pathways during the process of endothelial differentiation.

We used a similar approach to try to identify the underlying molecular mechanisms involved at various stages of endothelial differentiation. We first evaluated the genes that are upregulated in CD144 positive cells at day 3.5 (angioblast) compared to the hemangioblast. The transcription factors that are highly enriched in this population include KLF2, Sox11 and the forkhead box P1 transcription factor. Some of the potential networks and pathways were also evaluated using the PathwayAssist program (Additional files 4 and 5). Similar analyses were conducted using the Pathway Assist program for genes identified to be enriched in CD144 positive cells (ES cell day 6.5) compared to day 2.5 VEGF-R2 positive ES cells (Additional files 6 and 7) and for CD144 positive cells isolated from mouse embryos at day 9.5, which is comparable to day 6.5 in the ES cell differentiation system, compared to CD144 negative cells (Additional files 8 and 9). We recognize that there are limitations of this type of analysis, and that the results of this analysis are only suggestive of potential pathways among the identified genes and transcription factors, and these analyses are therefore presented as supplemental data. Future studies will be needed to verify specific aspects of the putative pathways identified.

## Discussion

### Overlap between hematopoietic and endothelial lineages

The close association of endothelial and hematopoietic lineage has now been demonstrated in several studies in a wide variety of species including xenopus, mice, and humans. Our studies similarly support a very close temporal and spatial expression pattern of endothelial and hematopoietic progenitor cells in differentiating ES cells. In human embryonic tissues, a population of endothelial-like cells with endothelial and hematopoietic potential expressing VEGF-R2, VE-cadherin, but not CD45, was similarly identified [26]. We observed weak VE-cadherin expression on CD41 positive cells in both the yolk sac and differentiating EBs, but did not observe CD41 expression on endothelial cells lining the vascular channels in the EB or yolk sac, suggesting that as CD41 expression occurs the cells bud off of the developing vasculature.

### Differentiation of the hemangioblast toward the hematopoietic lineage

Several transcription factors have been shown to be critical for different stages of hematopoietic cell differentiation. Some of the critical transcription factors known to play a role early in hematopoietic cell differentiation include Runx-1 (AML-1), GATA-1, and members of the Ikaros zinc finger transcription factor family. We also observed enrichment for these factors in early CD41 positive cells within differentiating EBs based on our microarray data. Although the EB is generally considered as a model of primitive hematopoiesis, similar to what is observed in the yolk sac, the expression of many Ikaros family members that are known to regulate several different hematopoietic lineages suggest a possible broader role for the CD41 positive cells identified in developing EBs in several hematopoietic lineages [27].

### Differentiation of the hemangioblast toward the endothelial lineage

Considerably less is known about the transcription factors and molecular mechanisms involved in regulating the differentiation of the hemangioblast along the endothelial lineage. In early (day 3.5) CD144 positive cells we observed that the increased expression of genes involved in regulating endothelial function such as Tie1 and the endothelin receptor B, compared to genes expressed in VEGF-R2 positive hemangioblast cells (day 2.5). We similarly identified a select number of transcription factors that were enriched in these cells, including KLF2, Sox11, and forkhead box (Fox) P1. Targeted disruption of FOXP1, which is expressed in the endocardium of the heart, is associated with abnormalities in cardiac development, similar to that observed with NFATc1 [28]. The Krueppel-like factor (KLF) family members have also been shown to be involved in cellular differentiation. KLF2 is expressed in the endothelium and is upregulated in response to fluid shear stress [29,30].

Because the early CD144 positive cells may not represent fully differentiated endothelial cells, microarray analysis was also performed on CD144 positive cells at a later stage of differentiation in the EBs (day 6.5) and isolated from the embryo (day 9.5). In addition to increased expression of several Sox family members (11 and 17), the expression of several other transcription factors including the nuclear hormone receptor COUP-TFII (NR2F1), MEF2C, and KLF2, were observed. Targeted disruption of the orphan nuclear receptor COUP-TFII results in abnormal vascular and cardiac development [31]. One of the main downstream targets of COUP-TFII is the angiogenic factor Angiopoietin-1. COUP family members are involved in regulating the expression of BMP-4 during embryogenesis. BMP-4 promotes hematopoiesis and angiogenesis during vascular development [32].

In CD144 expressing cells of the embryo (day 9.5) we observed significant overlap in the endothelial-specific genes and transcription factors upregulated in the CD144 positive cells derived from the embryo and differentiating EBs at day 6.5. The transcription factors that were highly expressed and enriched in these cells included, Sox 7, Sox 17, MEF2C, and KLF2. In addition several other transcription factors were also identified in CD144 positive cells of the embryo including the ETS transcription factor family members ERG and ELK3, GATA2, NFATc1, Sox18, Forkhead O1A, and C/EBP delta. The Ets transcription factor ELK3 is highly expressed at sites of vasculogenesis and angiogenesis during early mouse development [33]. Furthermore, down-regulation of ELK3 is associated with marked reductions in VEGF expression. Although ELK3 principally functions as a repressor, Ras activation leads to a phosphorylated form of ELK3 that is highly active [33]. The Ets factor ERG is also enriched in endothelial cells and down regulation of ERG in endothelial cells is associated with reductions in the expression of the endothelial-specific genes [34]. The GATA factors have similarly been shown to regulate endothelial-specific genes. GATA2 is involved in the regulation of PECAM-1, endothelin-1, and ICAM-2 [35-37]. Both the forkhead family members and NFATc1 function as transcriptional mediators of VEGF in endothelial cells [38,39]. There are no previous reports suggesting a role for C/EBP delta in endothelial function or gene expression.

Microarray chip analysis has been used in a number of studies for uncovering genes that are differentially expressed during hematopoietic and endothelial differentiation (see Table 8). In most of these studies, differentiating mouse or human ES cells have been used as a model system for hematopoietic/endothelial differentiation. Isolation of the VEGF-R2^+ ^cell population at an early stage of ES cell differentiation was done in all of the mouse ES cells studies as an already established population with hemangioblast properties [41-43,45-47]. Human ES cell hemangioblasts were more tightly defined by sorting out the VEGFR-2^+^/CD34^+ ^or VEGFR-2^+^/CD31^+ ^cells [38]. A number of mesodermal markers were identified in the VEGFR-2^+ ^cells at D2.5 of differentiation including Brachyuary [37,40], msh homeo box homolog 1 (Msx1), NODAL, inhibin BA/BB [37], Hand1, Mesp1 [40], and canonical Wnt-signalling associated genes such as Wnt3a and Wnt8a [42]. These genes are involved in processes such as development, transcription, organ and system development, with genes such as Sox9, HoxB2, and HoxB3 being specifically involved in hemangioblast differentiation [40].

**Table 8:** Microarray Studies of Hematopoietic (HP) and Endothelial Cell (EC) Differentiation in EBs

**Study**	**Species**	**Markers**	**Differentiation**	**Modulators**
1. Chen D et al[40]	Human	Whole EBs	HP	
2. Woll PS et al[41]	Human	CD31, CD34, Flk-1	HP/EC	
3. Schenke K et al[42]	Human	Whole ES	HP/EC	Col IV, FGF4
4. Lu SJ et al[43]	Human	Whole EB/ES	EC	VEGF, BMP4
5. Wang H et al[44]	Mouse	VEGF-R2	EC	Wnt
6. Williamson AJ et al[45]	Mouse	Bry/VEGF-R2	HP	
7. Rolny C et al[46]	Mouse	CD31, VEGF-R2	EC	
8. Wang C. et al[47]	Mouse	Whole EBs	HP/EC	Lycat
9. Wu Y. et al[48]	Mouse	VEGF-R2	EC	PRDM6
10. Ferguson JE et al[49]	Mouse	VEGF-R2	EC	ASB4
11. Ng YS et al[50]	Mouse	Whole EBs	VEGF	

During the hemangioblast phase a number of early hematopoietic and endothelial markers are found to be co-expressed on the VEGFR-2^+ ^cells from day 3–6 of differentiation. Genes found in these group include CD34, TAL/SCL, Runx1/AML1, GATA-2/3, Lmo2, Notch1, c-kit, CD41, HoxB4 (all of them referred to as hematopoietic commitment markers), and genes thought to be expressed on both, hematopoietic and endothelial progenitors including CD31, VE-cadherin, CXCRF, Flt-1 [[37,38] and [41]], KDR [40], TGFα-1, Bmp7, Smad1, Wnt associated genes FrzB, β-catenin, N-cadherin, Msx2, Cyclin D1, Dkk1 [41], lysocardiolipin acyltransferase (Lycat) [44] and finally PRDM6, encoding a Krueppel-like zinc finger protein (KLF) that functions in cell-fate decisions and malignant transformation [45]. Most of these genes are downregulated as the divergence of the two cell lines takes place, however, a number of these markers maintain steady levels of expression throughout the course of differentiation. The onset of the definitive hematopoiesis is marked by the expression of CD45, the genes involved in adult hematopoietic cell renewal (PIK3R1, MDR1, IRS1, RGS18, SUMO-1 and Wnt5) [37], CXCL12, Alcam [39], mayeloblastosis oncogene (Myb) and Hemoglobin genes [43].

Genes whose expression is enriched in more mature endothelial cells from day 6 and onwards include VE-cadherin, CXCR4 [38], Angiopoietin 2 (Ang2), Endothelin 1 (End1) [39], ICAM-2, neuropilin-1, endoglin, transforming growth factor genes (TGFβ-2, BMP4 and BMPR-1), Wnt associated genes (Wnt2, Wnt5a, Nkd1, cyclin D2, Frzd7, Lef1, Frzb, Sox17, Msx2, fibrinogen, Myc) [41], Tie-2, genes involved in angiogenic response to VEGF such as PDGFBB, PDGFR-1, Shb [43], ASB4 (which functions in vascular differentiation in an oxygen-dependent manner) [46], caveolin, Hey1, FGFR-1, and adhesion protein genes cadherin 5 and claudin 3 [47]. The findings from our microarray studies are in accord with the previously published studies. In addition, we have identified several additional genes such as Ikaros and Helios zinc-finger transcription factors, Erg, claudin 5, and Sox17, that segregate with the hematopoietic or endothelial cell populations (see Table 1,2,3,4,5,6,7). Future studies will be needed to identify whether or not these genes play a significant role as determinants of endothelial or hematopoietic cell differentiation.

## Conclusion

In conclusion, the results of our study support the use of embryonic stem cells to study the molecular events underlying hematopoiesis and endothelial differentiation. Our results demonstrate the expression of distinct sets of genes in populations of embryonic stem cells expressing CD41 or CD144 that are consistent with genes associated with the hematopoietic or endothelial lineages. Future studies should be directed at defining the role of selected transcription factors that are enriched in selected subsets of stem cells during the process of differentiation.

## Authors' contributions

VN–K carried out the stem cell differentiation, flow cytometry, and immunohistochemistry studies. She also contributed to the writing and editing of the manuscript. MB contributed to the microarray analysis and interpretation, and writing of the manuscript. HHO contributed to the microarray analysis. TL contributed to the design of the microarray studies. PO contributed to the design of all the experiments, and the writing and editing of the manuscript. All authors read and approved the final manuscript.

## Supplementary Material

Additional file 1 **A comparative analysis of genes that are upregulated during the stages of endothelial differentiation in the embryoid body or embryo**. Forty one genes (Section VI) are upregulated at the angioblast stage (day 3.5, CD144-positive), during later stages of endothelial differentiation in the embryoid body (day 6.5, CD144-positive) and in CD144-positive cells of the embryo at day 9.5. Section IV depicts the 139 genes that are enriched in CD144-positive cells of the embryoid body at early and later stages (day 3.5 and 6.5), and Section V depicts genes that are enriched in CD144-positive cells in later stages of the embryoid body (day 6.5) and in CD144-positive cells of the embryo at day 9.5. Click here for file

Additional file 2 **Pathway analysis of transcription factors enriched in CD41-positive cells**. Pathway analysis of selected transcription factors identified in CD41-positive cells at day 3.5 in the developing embryo were compared to those identified VEGF-R2-positive cells at day 2.5. Click here for file

Additional file 3 **Pathway analysis of genes enriched in CD41-positive cells**. Pathway analysis for the extended gene list identified in CD41-positive cells at day 3.5 in the developing embryo were compared to those identified VEGF-R2-positive cells at day 2.5. The networks predicted by the program PathwayAssist are shown. Click here for file

Additional file 4 **Pathway analysis of transcription factors that are upregulated in early CD144 positive cells**. Pathway analysis of selected transcription factors identified in CD144-positive cells at day 3.5 (angioblast) compared to those identified VEGF-R2-positive cells at day 2.5. The networks predicted by the program PathwayAssist are shown. Click here for file

Additional file 5 **Pathway analysis of genes that are upregulated in early CD144 positive cells differentiation**. Pathway analysis for extended gene list identified in CD144-positive cells at day 3.5 (angioblast) compared to those identified VEGF-R2-positive cells at day 2.5. The networks predicted by the program PathwayAssist are shown. Click here for file

Additional file 6 **Pathway analysis of transcription factors involved later stages of endothelial differentiation in ES cells**. Pathway analysis of transcription factors upregulated in CD144-positive cells at a later stage of ES cell differentiation (day 6.5) were compared to the VEGF-R2-positive cells at day 2.5. The networks predicted by the program PathwayAssist are shown. Click here for file

Additional file 7 **Pathway analysis of transcription factors involved later stages of endothelial differentiation in ES cells**. Pathway analysis of extended gene list upregulated in CD144-positive cells at a later stage of ES cell differentiation (day 6.5) were compared to the VEGF-R2-positive cells at day 2.5. The networks predicted by the program PathwayAssist are shown. Click here for file

Additional file 8 **Pathway analysis of transcription factors that are upregulated in CD144-positive versus CD144-negative cells in E9.5 embryos**. Pathway analysis of transcription factors upregulated in CD144-positive cells that were isolated from mouse embryos at day 9.5 were compared to CD144-negative cells. The networks predicted by the program PathwayAssist are shown. Click here for file

Additional file 9 **Pathway analysis of the extended gene list that are upregulated in CD144-positive versus CD144-negative cells in E9.5 embryos**. Pathway analysis of genes upregulated in CD144-positive cells that were isolated from mouse embryos at day 9.5 were compared to CD144-negative cells. The networks predicted by the program PathwayAssist are shown. Click here for file
