# Supplementary figures and images for: Gene expression analysis of embryonic stem cells expressing VE-cadherin (CD144) during endothelial differentiation
Source: BMC Genomics. 2008 May 22;9:240. doi: 10.1186/1471-2164-9-240 (PMC2440556; doi:10.1186/1471-2164-9-240)

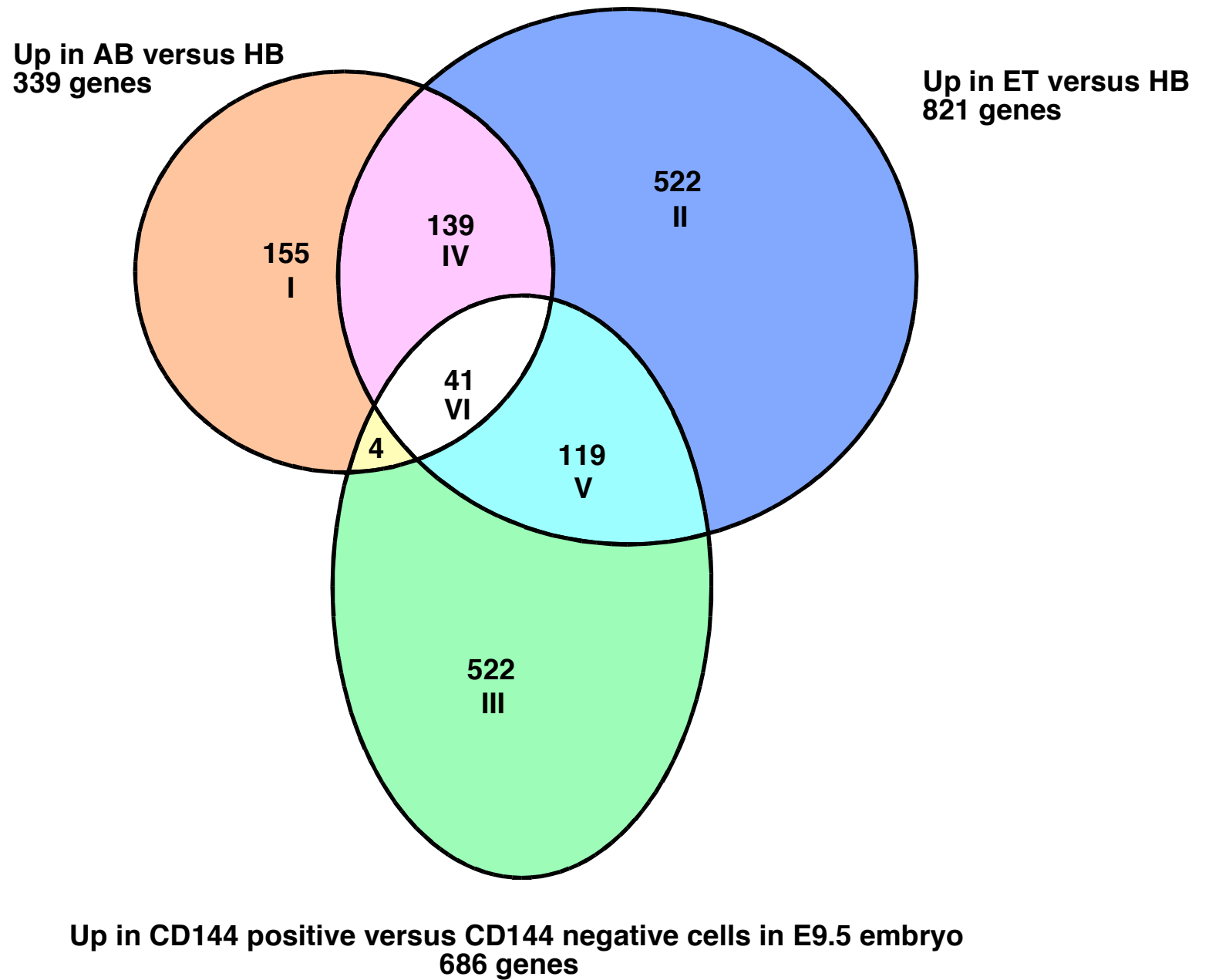

**Supplemental Figure 1**

Supplement: Additional file 1 — A comparative analysis of genes that are upregulated during the stages of endothelial differentiation in the embryoid body or embryo. Forty one genes (Section VI) are upregulated at the angioblast stage (day 3.5, CD144-positive), during later stages of endothelial differentiation in the embryoid body (day 6.5, CD144-positive) and in CD144-positive cells of the embryo at day 9.5. Section IV depicts the 139 genes that are enriched in CD144-positive cells of the embryoid body at early and later stages (day 3.5 and 6.5), and Section V depicts genes that are enriched in CD144-positive cells in later stages of the embryoid body (day 6.5) and in CD144-positive cells of the embryo at day 9.5. [file 1471-2164-9-240-S1.pdf]

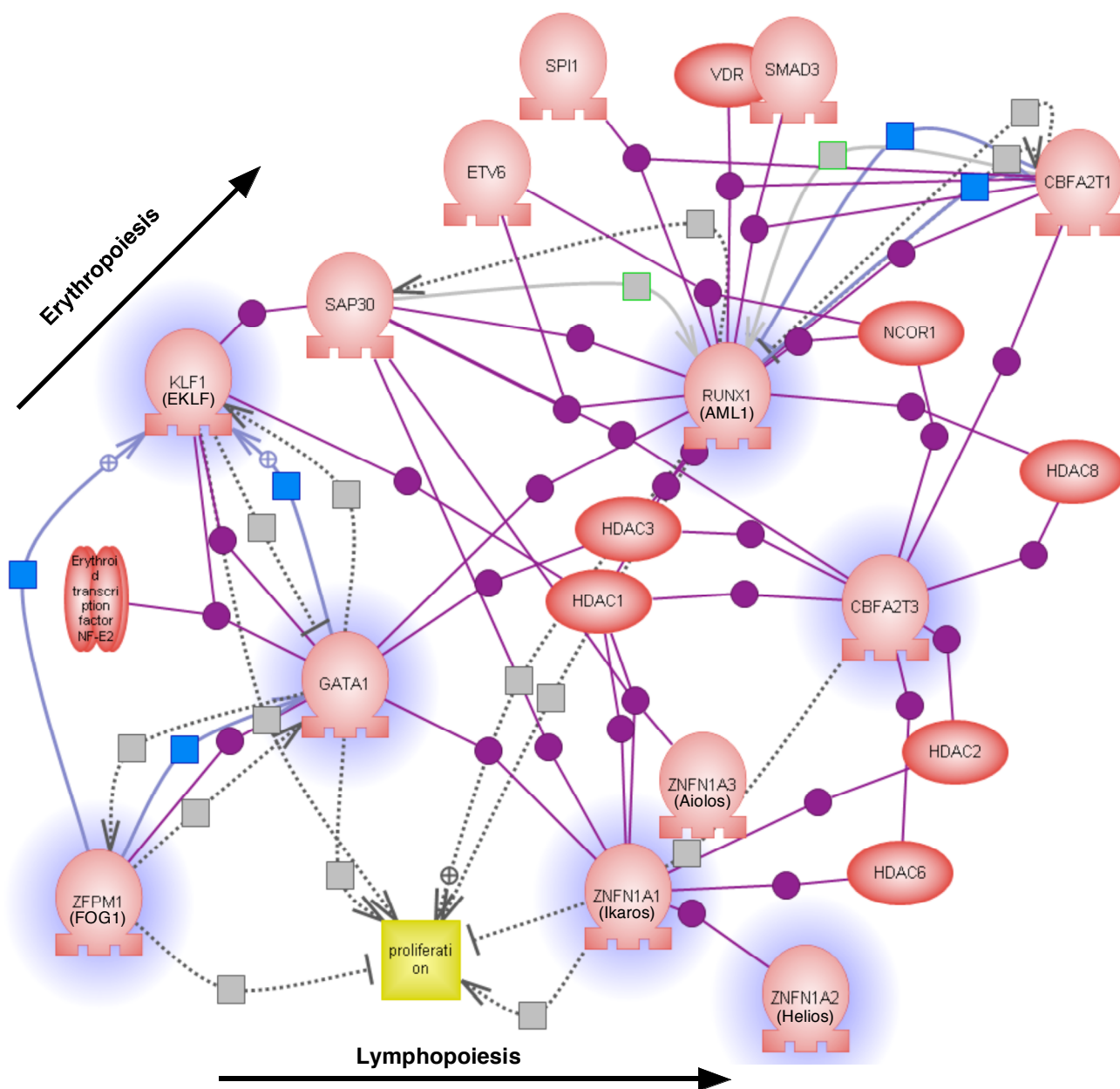

**Supplemental Figure 2**

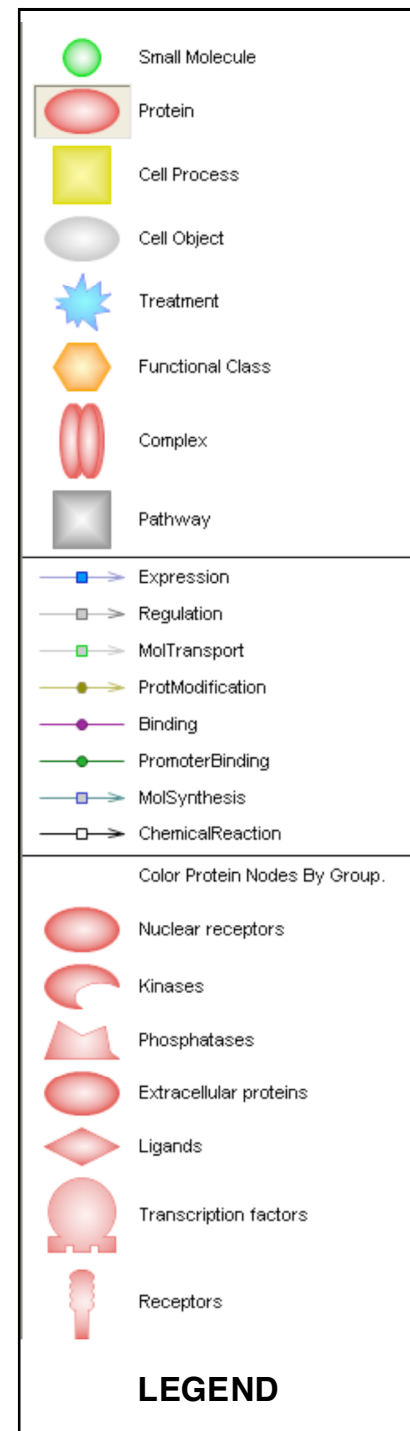

Supplement: Additional file 2 — Pathway analysis of transcription factors enriched in CD41-positive cells. Pathway analysis of selected transcription factors identified in CD41-positive cells at day 3.5 in the developing embryo were compared to those identified VEGF-R2-positive cells at day 2.5. [file 1471-2164-9-240-S2.pdf]

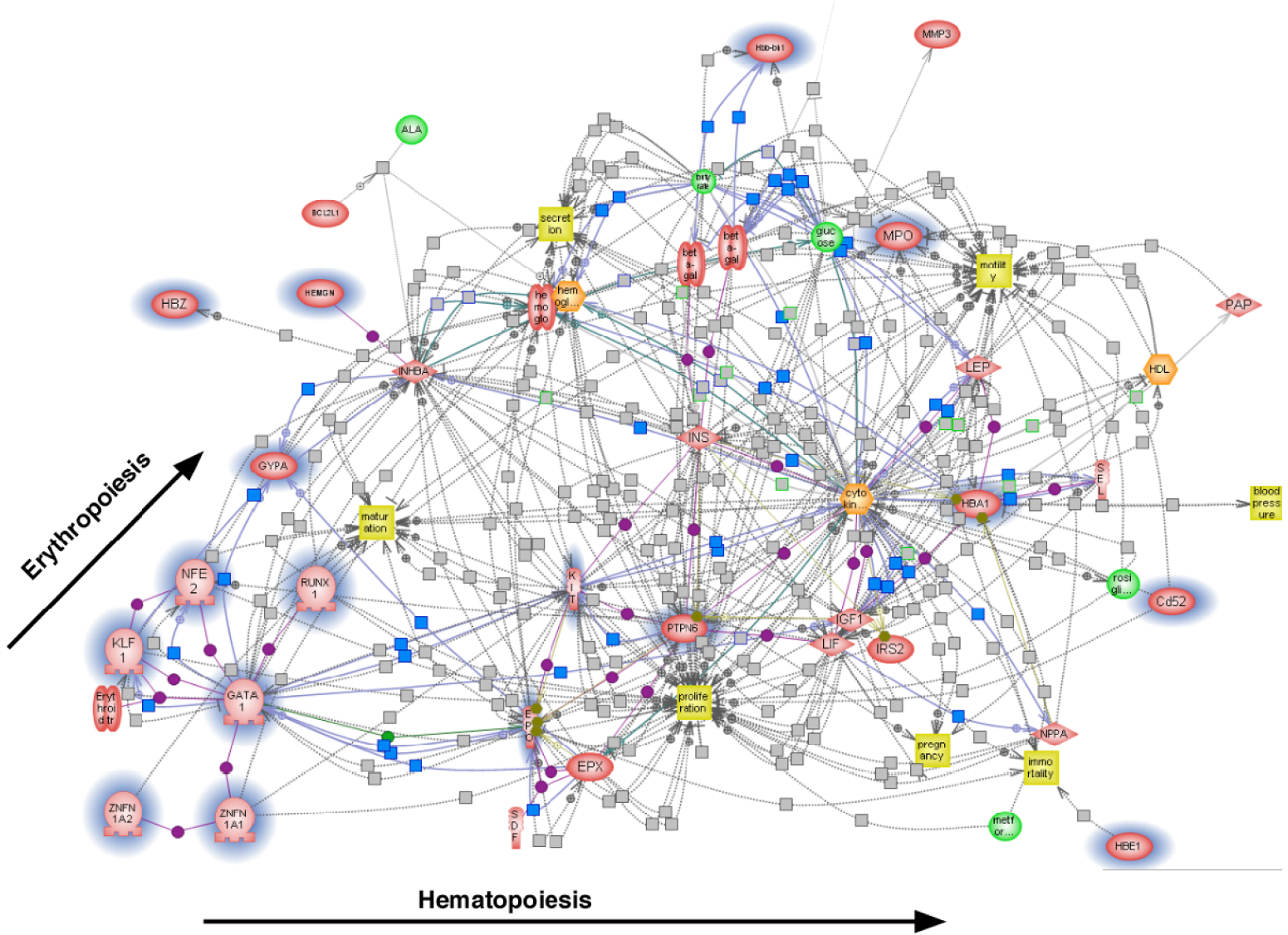

Supplement: Additional file 3 — Pathway analysis of genes enriched in CD41-positive cells. Pathway analysis for the extended gene list identified in CD41-positive cells at day 3.5 in the developing embryo were compared to those identified VEGF-R2-positive cells at day 2.5. The networks predicted by the program PathwayAssist are shown. [file 1471-2164-9-240-S3.pdf]

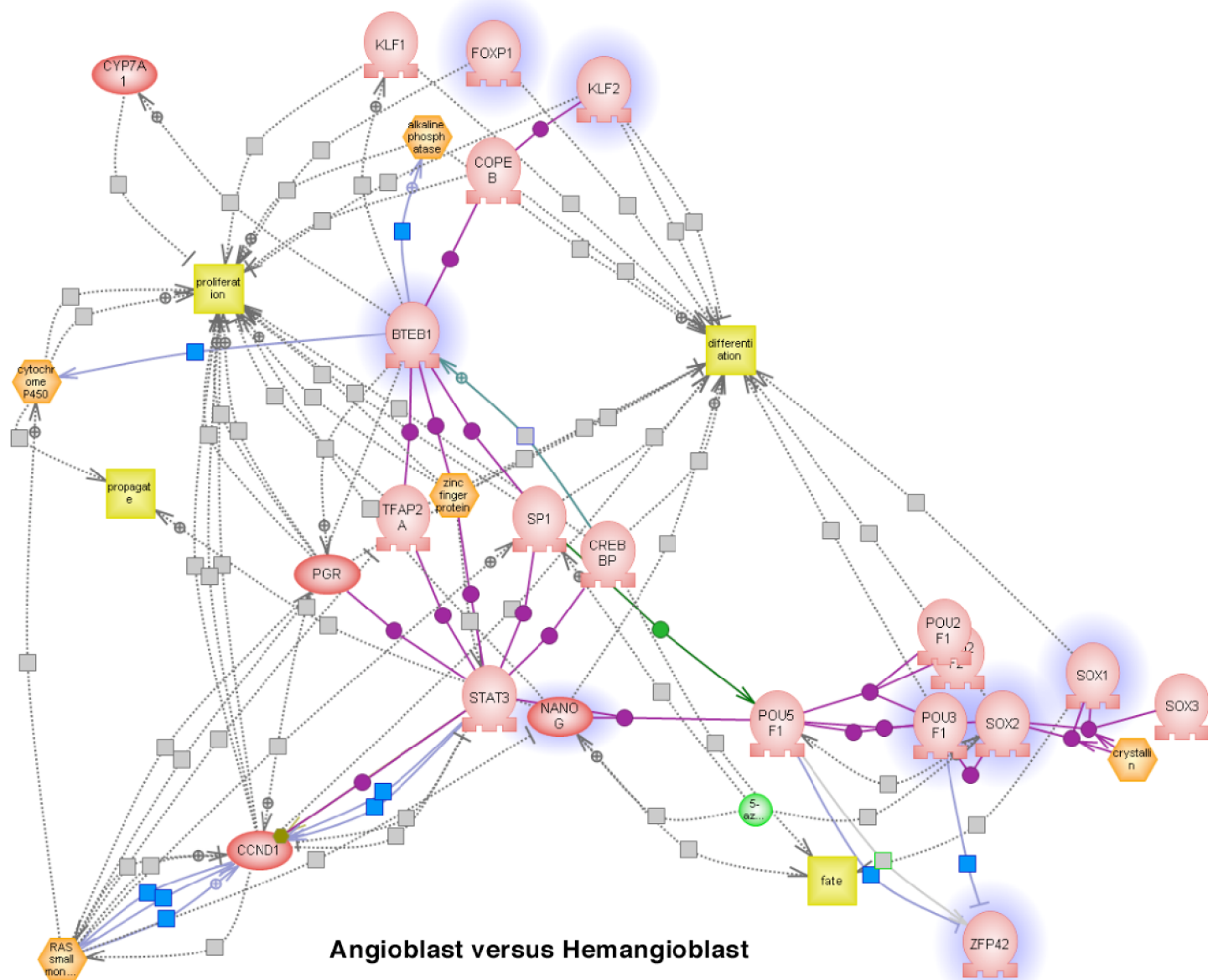

**Angioblast versus Hemangioblast**  
**Supplemental Figure 4**

Supplement: Additional file 4 — Pathway analysis of transcription factors that are upregulated in early CD144 positive cells. Pathway analysis of selected transcription factors identified in CD144-positive cells at day 3.5 (angioblast) compared to those identified VEGF-R2-positive cells at day 2.5. The networks predicted by the program PathwayAssist are shown. [file 1471-2164-9-240-S4.pdf]

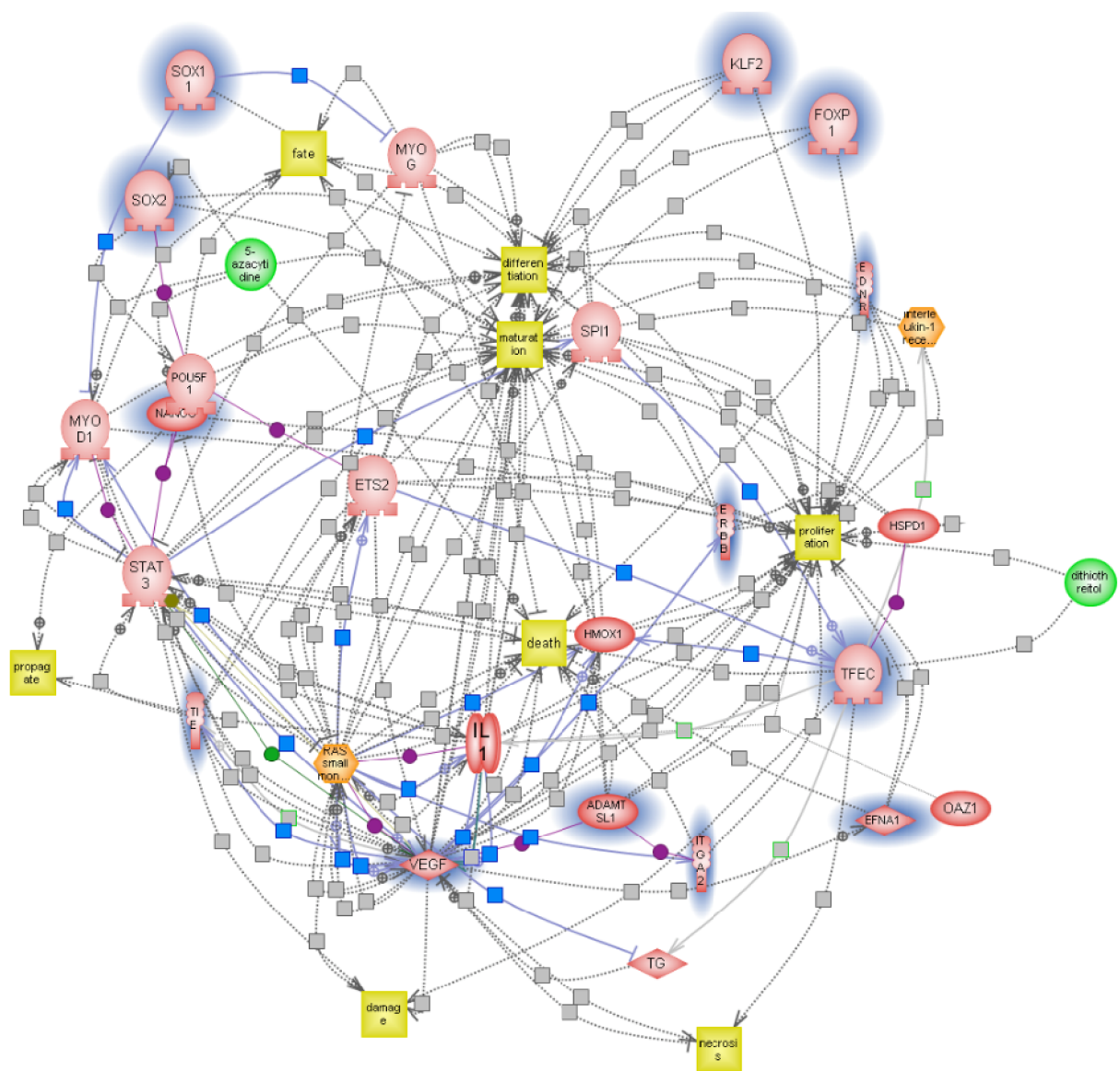

**Angioblast versus Hemangioblast**

**Supplemental Figure 5**

Supplement: Additional file 5 — Pathway analysis of genes that are upregulated in early CD144 positive cells differentiation. Pathway analysis for extended gene list identified in CD144-positive cells at day 3.5 (angioblast) compared to those identified VEGF-R2-positive cells at day 2.5. The networks predicted by the program PathwayAssist are shown. [file 1471-2164-9-240-S5.pdf]

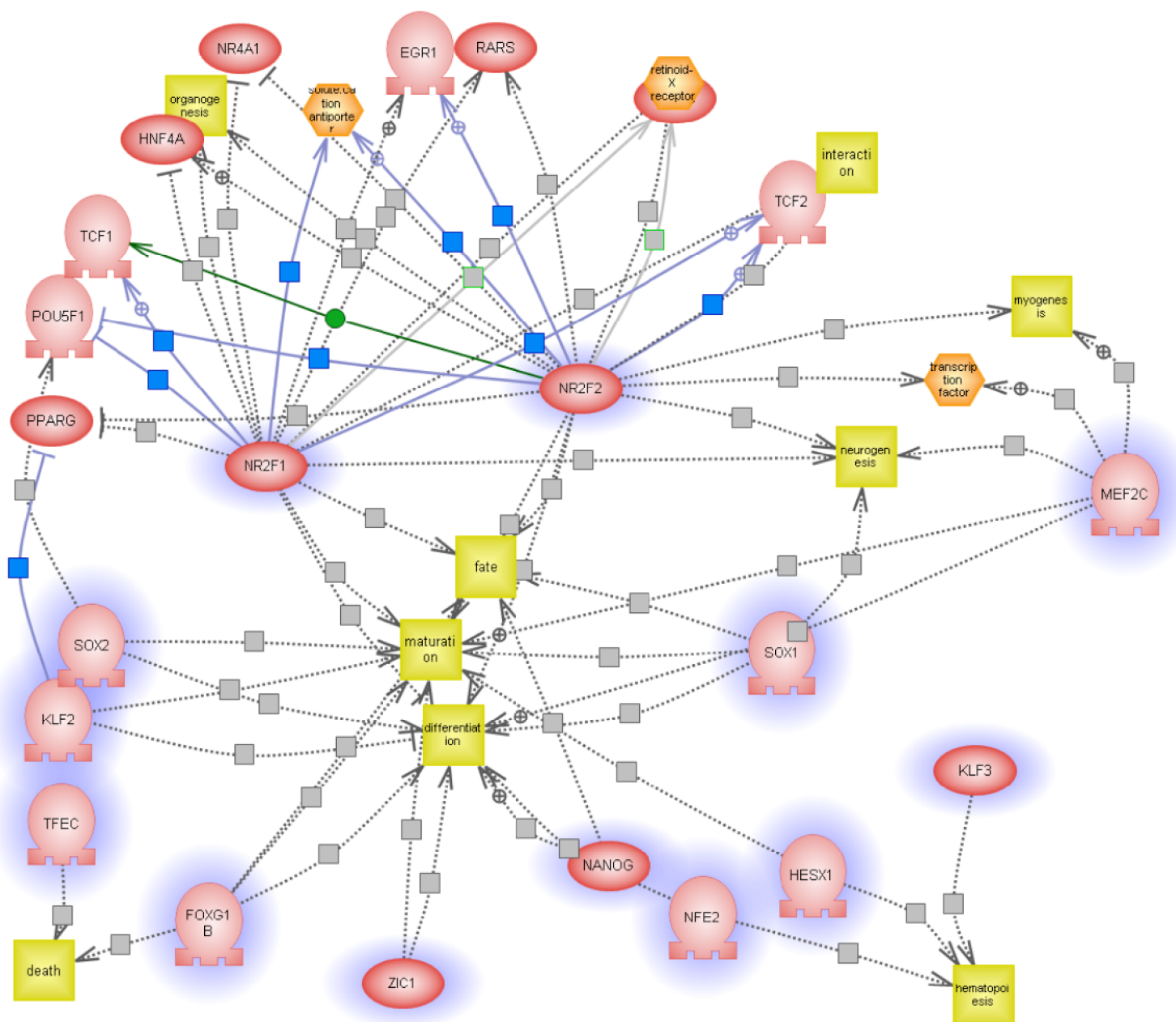

**Day 6.5 Endothelial cell versus Hemangioblast**

**Supplemental Figure 6**

Supplement: Additional file 6 — Pathway analysis of transcription factors involved later stages of endothelial differentiation in ES cells. Pathway analysis of transcription factors upregulated in CD144-positive cells at a later stage of ES cell differentiation (day 6.5) were compared to the VEGF-R2-positive cells at day 2.5. The networks predicted by the program PathwayAssist are shown. [file 1471-2164-9-240-S6.pdf]

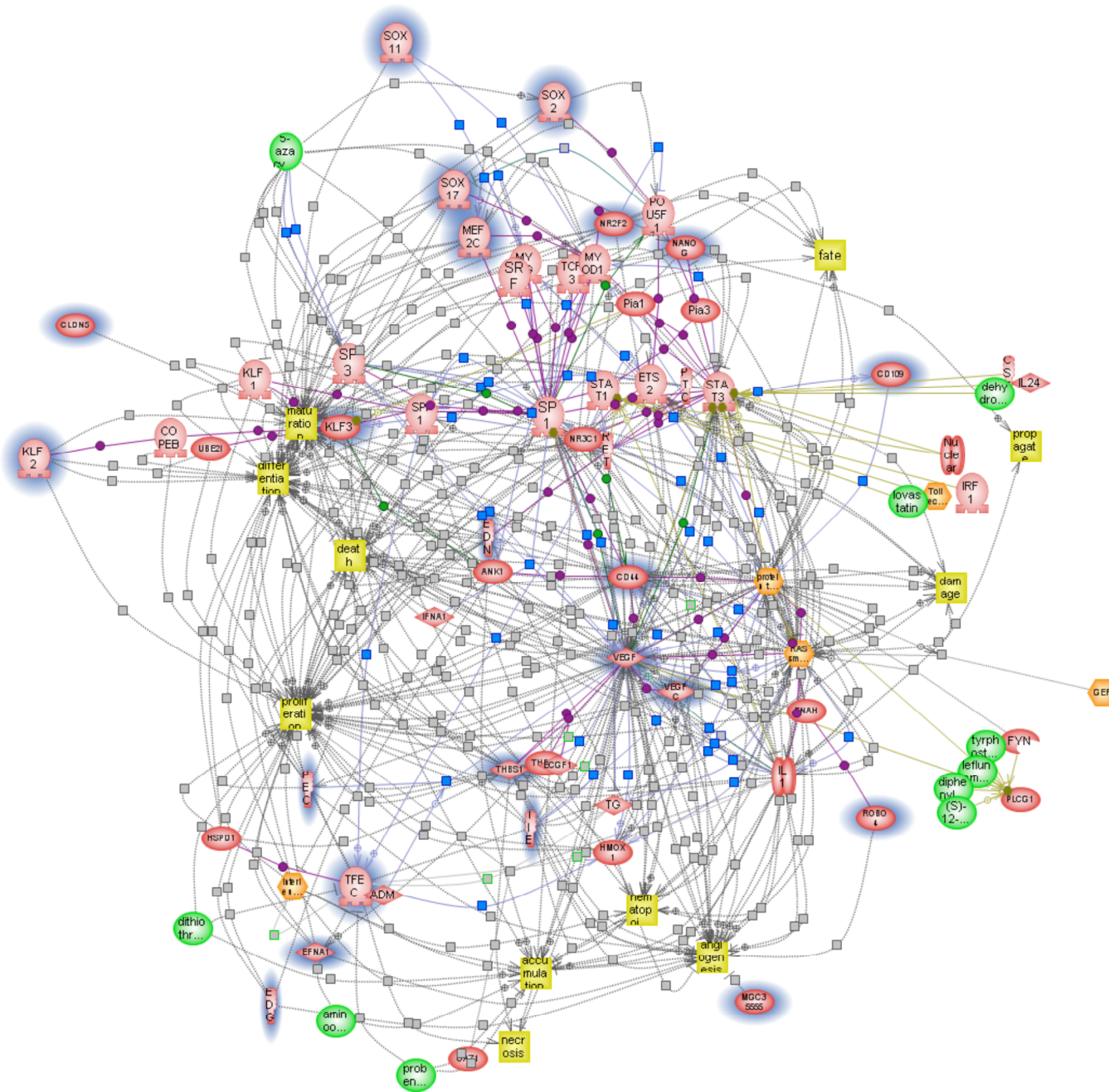

Day 6.5 Endothelial Cell versus VEGF-R2 positive Day 2.5 Hemangioblast

Supplemental Figure 7

Supplement: Additional file 7 — Pathway analysis of transcription factors involved later stages of endothelial differentiation in ES cells. Pathway analysis of extended gene list upregulated in CD144-positive cells at a later stage of ES cell differentiation (day 6.5) were compared to the VEGF-R2-positive cells at day 2.5. The networks predicted by the program PathwayAssist are shown. [file 1471-2164-9-240-S7.pdf]

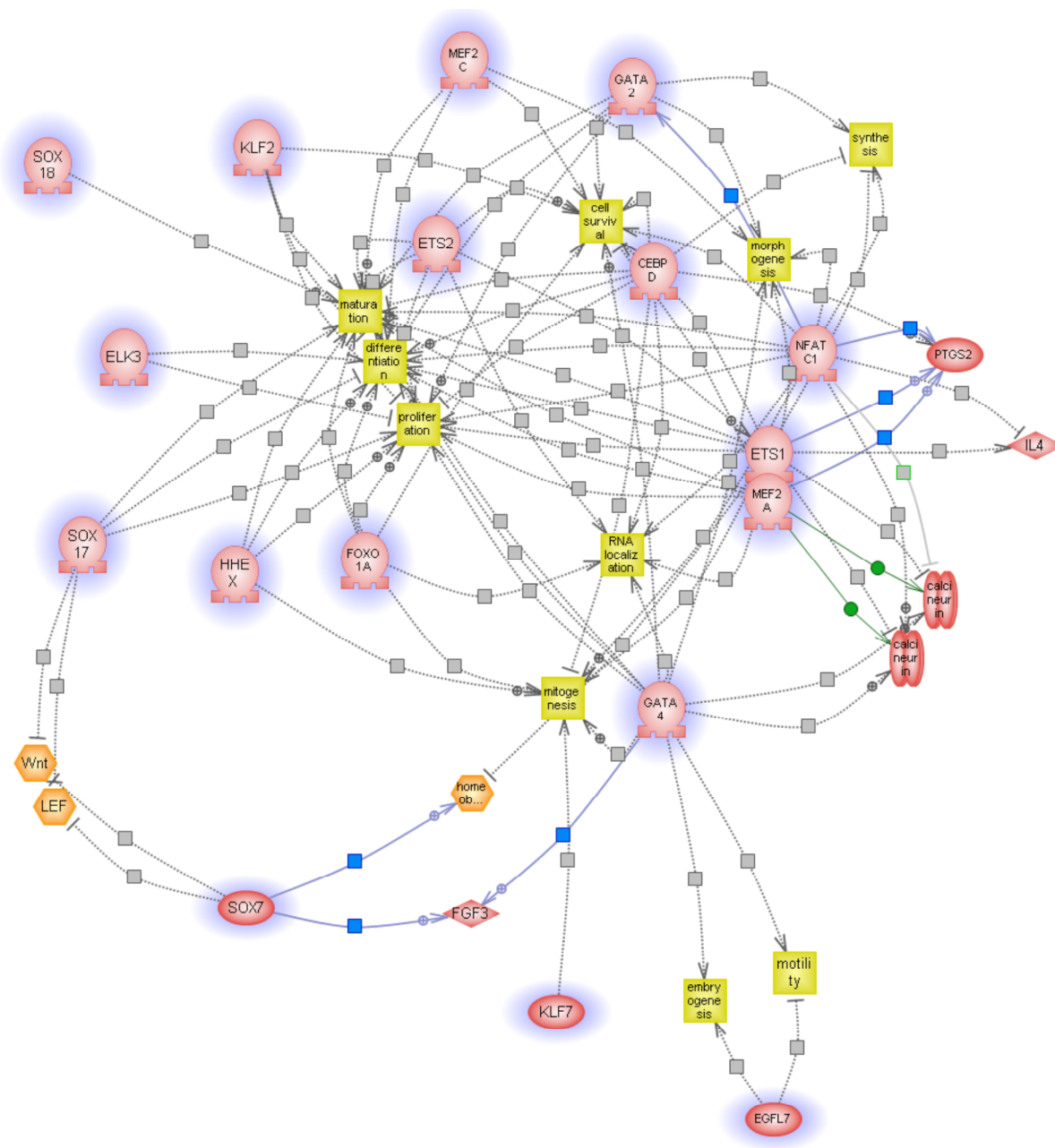

**CD144 positive versus negative**

**Supplemental Figure 8**

Supplement: Additional file 8 — Pathway analysis of transcription factors that are upregulated in CD144-positive versus CD144-negative cells in E9.5 embryos. Pathway analysis of transcription factors upregulated in CD144-positive cells that were isolated from mouse embryos at day 9.5 were compared to CD144-negative cells. The networks predicted by the program PathwayAssist are shown. [file 1471-2164-9-240-S8.pdf]
