# Supplementary material for: Gene expression analysis of embryonic stem cells expressing VE-cadherin (CD144) during endothelial differentiation
Source: BMC Genomics. 2008 May 22;9:240. doi: 10.1186/1471-2164-9-240 (PMC2440556; doi:10.1186/1471-2164-9-240)
Supplement: Additional file 9 — Pathway analysis of the extended gene list that are upregulated in CD144-positive versus CD144-negative cells in E9.5 embryos. Pathway analysis of genes upregulated in CD144-positive cells that were isolated from mouse embryos at day 9.5 were compared to CD144-negative cells. The networks predicted by the program PathwayAssist are shown. [file 1471-2164-9-240-S9.pdf]

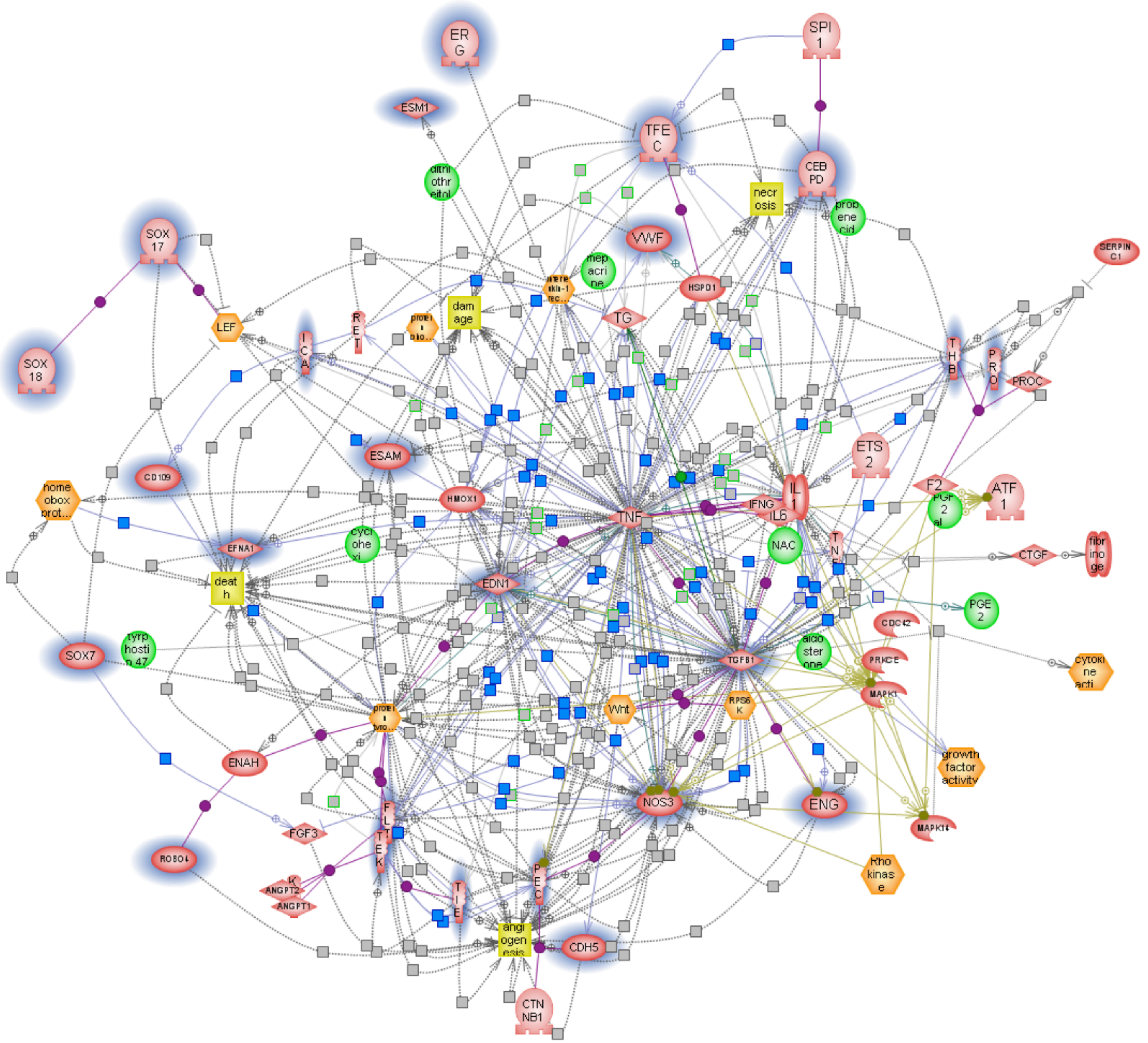

CD144 postivie versus CD144 negative

Supplemental Figure 9
